# Sex differences in testosterone and obesity

**DOI:** 10.3389/fmed.2026.1992734

**Published:** 2026-10-28

**Authors:** Saki Komai, Takahiro Tsutsumi, Kyoichiro Tsuchiya

**Affiliations:** Department of Diabetes and Endocrinology, Graduate School of Interdisciplinary Research, Faculty of Medicine, University of Yamanashi, Chuo, Yamanashi, Japan

**Keywords:** androgen receptor, hypogonadism, obesity, polyendocrine metabolic ovarian syndrome, sex differences, testosterone

## Abstract

Androgens, particularly testosterone, play a central role in the regulation of energy metabolism and adipose tissue distribution; however, their physiological effects exhibit marked sexual dimorphism. In males, testosterone, primarily secreted by the testes, has been reported to inhibit adipogenesis and limit the accumulation of visceral fat. Conversely, obesity itself may suppress testosterone production, creating a deleterious feedback loop between the two. In females, testosterone is derived from the ovaries, adrenal glands, and peripheral conversion of androgen precursors, and obesity may be associated with relative androgen excess. Excess androgen signaling is implicated in the promotion of further fat accumulation and is a characteristic feature of metabolic disorders such as polyendocrine metabolic ovarian syndrome (PMOS, formerly known as PCOS). This review summarizes the bidirectional relationship between testosterone and obesity in both sexes and discusses future research directions.

## 1 Introduction

Obesity is a pressing global health challenge, profoundly involved in the pathogenesis and progression of numerous chronic diseases, including type 2 diabetes, dyslipidemia, hypertension, cardiovascular disease, malignancy, and metabolic dysfunction-associated steatotic liver disease (MASLD) (1). Adipose tissue is no longer regarded merely as an energy storage depot but is recognized as an active endocrine organ that produces diverse bioactive molecules and communicates with other organs to regulate systemic metabolic homeostasis (2). In men, hypogonadism is closely associated with visceral fat accumulation, insulin resistance, metabolic syndrome, and type 2 diabetes. Conversely, obesity itself is associated with reduced circulating testosterone concentrations through both central and peripheral mechanisms, supporting a bidirectional relationship in which the two conditions may mutually exacerbate one another. Serum testosterone concentrations increase in association with the magnitude of weight loss, highlighting the importance of weight management in men with obesity-related functional hypogonadism (3–5). In contrast, most existing research on the relationship between testosterone and metabolism in females has focused on polyendocrine metabolic ovarian syndrome (PMOS), specifically addressing conditions underpinned by hyperandrogenism. Consequently, evidence regarding the interaction between testosterone and obesity in females remains less robust compared to that in males, and many aspects of its pathophysiology remain to be elucidated. Importantly, BMI does not fully reflect visceral fat, ectopic fat, or lean mass, and these adiposity phenotypes should be considered separately. Following a 2026 global consensus, PMOS was adopted to replace polycystic ovary syndrome (PCOS); because earlier guidelines and studies used PCOS, we use “PMOS (formerly PCOS)” at first mention and retain the terminology used in individual studies when relevant. In this review, we first provide an overview of the fundamental interactions between testosterone and adipose tissue. We then synthesize findings from basic and clinical research on the relationship between testosterone and obesity in both males and females. Furthermore, we discuss the current limitations in existing evidence and propose future research directions to address these unresolved clinical challenges. To improve transparency, we conducted a targeted PubMed search through September 2026 using combinations of terms related to testosterone, androgens, obesity, adipose tissue, body composition, insulin resistance, SHBG, hypogonadism, MOSH, PCOS/PMOS, and sex differences. Relevant reviews, guidelines, and original articles were selected based on their relevance to the scope of this review. The principal sex-specific differences in testosterone sources, obesity-related changes, underlying mechanisms, metabolic consequences, and remaining evidence gaps are summarized in Table 1.

**Table 1:** Sex-specific comparison of testosterone–obesity relationships.

Category	Men	Women
Testosterone sources	Testis: ∼95% Adrenal and peripheral sources: ∼5%	Premenopause Ovary: ∼25% Adrenal: ∼25% Peripheral conversion: ∼50% Postmenopause Ovary: ∼50% Adrenal: ∼10% Peripheral conversion: ∼40%
Obesity-driven mechanisms	Reduced SHBG lowers total testosterone Increased aromatization and leptin resistance suppress HPG axis activity Inflammation decreases HPG axis activity and Leydig cell steroidogenesis	Adipose dysfunction and inflammation drive insulin resistance Hyperinsulinemia increases ovarian androgen production and lowers SHBG Adipose AKR1C3 elevates local testosterone production
Metabolic consequences	Low testosterone levels in men are associated with: Insulin resistance Decreased lean mass	Androgen excess in PMOS is associated with: Central adiposity Insulin resistance Adipose dysfunction
Evidence base and gaps	Associations between low testosterone and adverse body composition are relatively well established Interventional studies support the body composition benefits of testosterone replacement therapy	Evidence is largely derived from PMOS and experimental models Metabolic effects of physiological testosterone ranges in women without PMOS remain limited

The table summarizes the principal sources of testosterone, obesity-related mechanisms, metabolic and body-composition consequences, and remaining evidence gaps in men and women. Evidence in women is largely derived from observational studies of PMOS and experimental models, whereas interventional studies in men have demonstrated improvements in body composition with testosterone replacement therapy. SHBG, sex hormone-binding globulin; HPG, hypothalamic–pituitary–gonadal; AKR1C3, aldo-keto reductase family 1 member C3; PMOS, polyendocrine metabolic ovarian syndrome.

## 2 Production and regulation of testosterone

### 2.1 Testosterone production in males

Testosterone is the primary androgen in males, with approximately 95% of circulating testosterone synthesized in testicular Leydig cells, while the remaining small fraction is derived from nontesticular sources, including adrenal androgen production and peripheral conversion of adrenal androgen precursors (6) (Figure 1). Testosterone production is tightly regulated by the hypothalamic–pituitary–gonadal (HPG) axis. Gonadotropin-releasing hormone, secreted in a pulsatile manner from the hypothalamus, stimulates the synthesis and secretion of luteinizing hormone and follicle-stimulating hormone from the anterior pituitary gland. Luteinizing hormone subsequently acts on testicular Leydig cells to stimulate testosterone production. Simultaneously, testosterone and its metabolite, estradiol, exert negative feedback on the hypothalamus and pituitary gland to maintain the homeostasis of the HPG axis (7). Most circulating testosterone is bound to sex hormone-binding globulin (SHBG) and albumin, whereas only 1%–2% remains unbound or free. The free and albumin-bound fractions are considered bioavailable testosterone because testosterone binds to albumin with relatively low affinity (8). In males, circulating testosterone concentrations vary markedly across the life course. Testosterone production increases during fetal development and again transiently during the first months after birth, a period known as “mini-puberty,” followed by low concentrations throughout childhood. With reactivation of the HPG axis at puberty, testosterone concentrations rise substantially and reach adult levels (9). Serum testosterone concentrations subsequently reach their highest levels during early adulthood and gradually decline with advancing age (10). In a prospective cohort study of healthy adult men followed for approximately 12 years, total testosterone decreased by 14.2%, whereas calculated free testosterone decreased more markedly by 19.1%, accompanied by a 3.0% increase in sex hormone-binding globulin. These changes persisted after adjustment for changes in body mass index, lifestyle factors, and intercurrent illness (11) (Figure 2).

**Figure 1:**
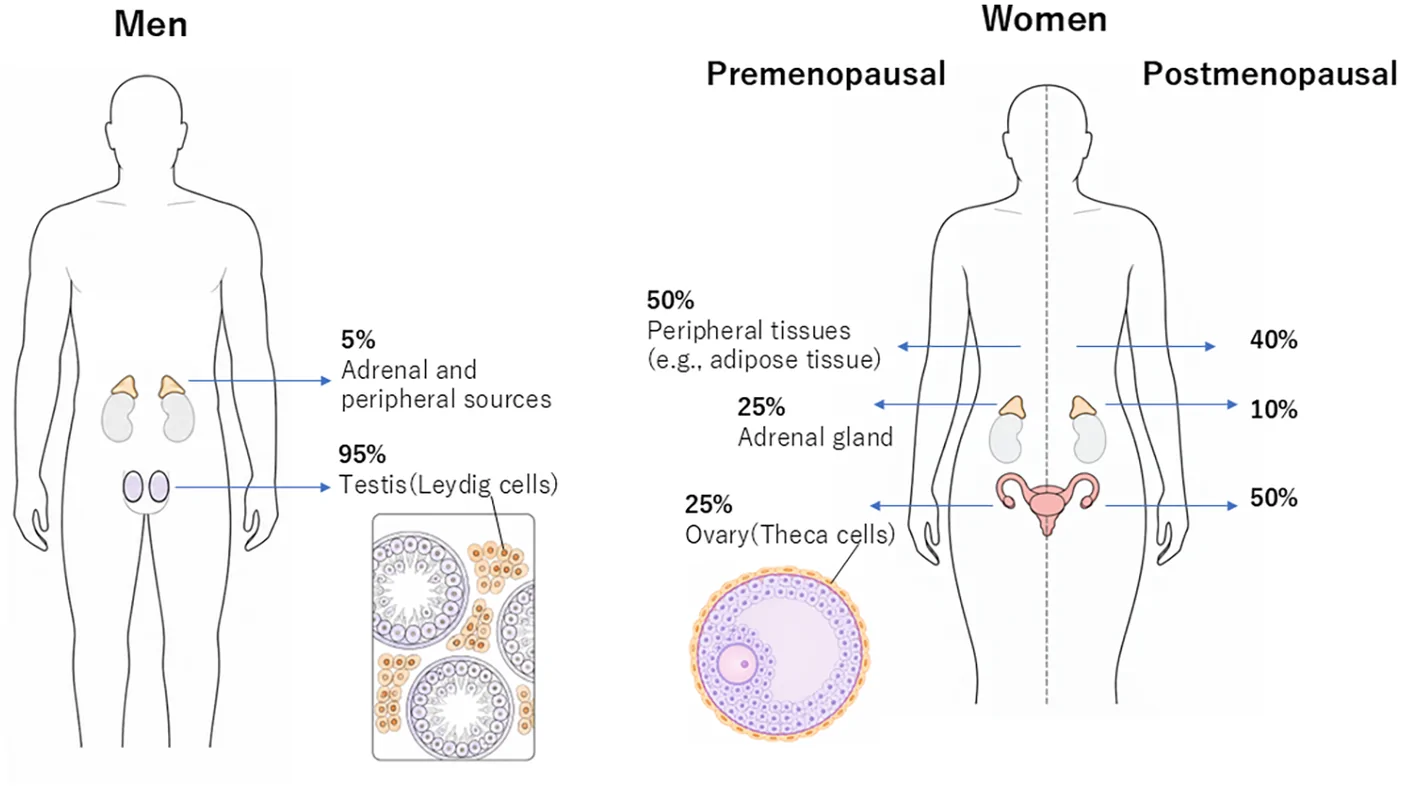
Sources of circulating testosterone in men and women. In men, approximately 95% of circulating testosterone is produced by testicular Leydig cells, with the remaining approximately 5% derived from adrenal and peripheral sources. In premenopausal women, approximately 25% of circulating testosterone is produced by the ovaries, 25% by the adrenal glands, and 50% through peripheral conversion of androgen precursors, including in adipose tissue. After menopause, the relative contribution of the ovaries increases to approximately 50%, while approximately 40% is derived from peripheral conversion and 10% from direct adrenal production. Percentages represent approximate contributions to circulating testosterone production.

**Figure 2:**
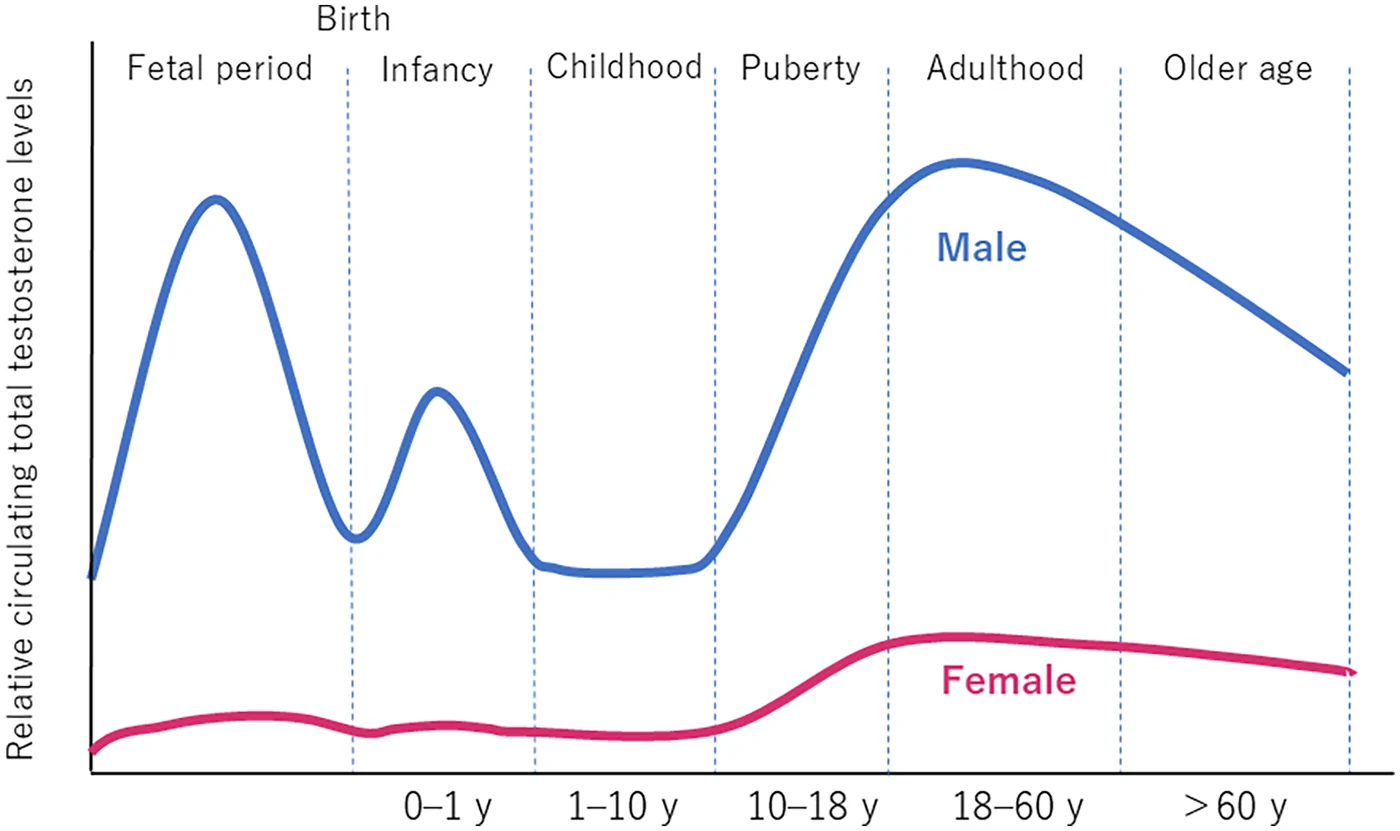
Schematic lifespan trajectories of circulating total testosterone in males and females. In males, testosterone levels rise during fetal life and infancy, remain low during childhood, increase markedly during puberty, peak in early adulthood, and gradually decline with age. In females, testosterone levels remain substantially lower, show no prominent postnatal surge, increase modestly with pubertal maturation, and decline gradually with age without an abrupt decrease at natural menopause. Curves are schematic and not to scale.

### 2.2 Testosterone production in females

Although testosterone is an important biologically active androgen in women, its sources are more diverse than in men. In women of reproductive age, approximately 25% of circulating testosterone is produced by the ovaries, 25% by the adrenal glands, and the remaining 50% in peripheral tissues through the conversion of circulating androstenedione (Figure 1). Thus, peripheral steroid conversion makes a substantial contribution to circulating testosterone in women (12). Circulating total testosterone concentrations are markedly lower in women than in men. A pooled analysis of studies using liquid chromatography–mass spectrometry reported 95% reference ranges of 7.7–29.4 nmol/L (approximately 222–848 ng/dL) in healthy young men aged 18–40 years and 0–1.7 nmol/L (approximately 0–49 ng/dL) in healthy menstruating women younger than 40 years (13). Luteinizing hormone stimulates androgen production in ovarian theca cells, whereas follicle-stimulating hormone induces aromatase expression in granulosa cells, thereby promoting the conversion of theca-derived androgens into estrogens (14). In females, the HPG axis is active during fetal life and is transiently reactivated after birth during the period known as “mini-puberty.” Although this postnatal activation occurs in both sexes, its hormonal pattern is sexually dimorphic, with follicle-stimulating hormone predominating in girls. Unlike in boys, a prominent postnatal testosterone surge is not observed; circulating testosterone concentrations decline rapidly after birth and reach the low levels characteristic of prepubertal girls by approximately the second week of life (15). Because most circulating testosterone is protein-bound, variations in SHBG can affect the interpretation of total testosterone concentrations in women. Total testosterone remains the primary biochemical measure, while SHBG and, when appropriate, calculated free testosterone may provide additional information. The free androgen index should be interpreted cautiously, particularly when SHBG concentrations are low (16). In adult women, circulating testosterone concentrations decline with advancing age, with a greater decline occurring during the earlier reproductive decades; however, natural menopause itself does not appear to exert an independent effect on androgen levels (17) (Figure 2). Importantly, the postmenopausal ovary remains hormonally active and continues to contribute to circulating testosterone concentrations (18). After natural menopause, approximately 50% of circulating testosterone is derived from the ovaries, approximately 40% from peripheral conversion of androstenedione, mainly of adrenal origin, and approximately 10% from direct adrenal production (12) (Figure 1).

## 3 Interaction between testosterone and obesity in males

### 3.1 Anti-obesity effects of testosterone

In males, experimental and interventional evidence suggests that testosterone may suppress the onset and progression of obesity through multiple mechanisms, acting on adipose tissue, skeletal muscle, and glucose metabolism (19). The mechanisms underlying the anti-obesity effects of testosterone are described below. First, testosterone directly inhibits adipocyte differentiation. Activation of the androgen receptor (AR) in preadipocytes suppresses the expression of peroxisome proliferator-activated receptor γ (PPARγ) and CCAAT/enhancer-binding protein α (C/EBPα), which are critical transcription factors for adipogenesis, thereby impeding differentiation into mature adipocytes (20, 21). Second, testosterone exerts anabolic effects on skeletal muscle and contributes to the maintenance and growth of skeletal muscle mass (19). Consistent with this effect, a systematic review and meta-analysis of randomized trials showed that testosterone supplementation increased lean body mass in middle-aged and older men (22). Furthermore, in clinical practice, it has been observed that men undergoing androgen deprivation therapy (ADT) for prostate cancer exhibit an increase in body fat mass, a decrease in lean body mass, and weight gain, demonstrating that androgen signaling plays a pivotal role in maintaining body composition (23).

### 3.2 Decline in testosterone associated with obesity

Obesity is strongly associated with reduced testosterone levels in men, and its mechanism involves multiple pathological processes. Consistent with this relationship, a recent cross-sectional study of Japanese men demonstrated inverse associations of total testosterone with both body mass index (BMI) and waist-to-hip ratio, and men with central obesity had significantly lower total and free testosterone concentrations than those without central obesity (24). Adipose tissue expresses aromatase (CYP19A1), which converts testosterone into estradiol. Increased adipose tissue mass may enhance peripheral aromatization and estradiol-mediated negative feedback on the HPG axis; however, the contribution of this pathway to obesity-related testosterone reduction in men remains incompletely established (3). Obesity-related reductions in SHBG contribute substantially to lower circulating total testosterone concentrations, although this mechanism only partially explains the observed testosterone reduction (3, 5). Obesity-associated alterations in leptin signaling may also contribute to HPG axis dysfunction. Leptin supports normal pulsatile GnRH secretion partly through hypothalamic kisspeptin neurons, and obesity-associated leptin resistance may impair this pathway, potentially contributing to reduced gonadotropin and testosterone secretion (3). In addition, chronic low-grade inflammation and metabolic disturbances associated with visceral obesity may impair HPG axis function. Pro-inflammatory cytokines, including tumor necrosis factor-α (TNF-α) and interleukin-6 (IL-6), may also directly suppress Leydig cell steroidogenesis, suggesting that both central and testicular mechanisms contribute to testosterone reduction in men with obesity (3, 25) (Figure 3). In addition, obstructive sleep apnea (OSA) is common in men with obesity and is associated with lower serum testosterone concentrations, particularly in severe OSA. Sleep fragmentation and nocturnal hypoxemia may contribute to impaired nocturnal testosterone secretion (26). The associations with both BMI and waist-to-hip ratio also indicate that regional fat distribution may provide information beyond BMI alone. Most human evidence for these pathways is observational; therefore, the proposed mechanisms should not be interpreted as proof of a single causal sequence.

**Figure 3:**
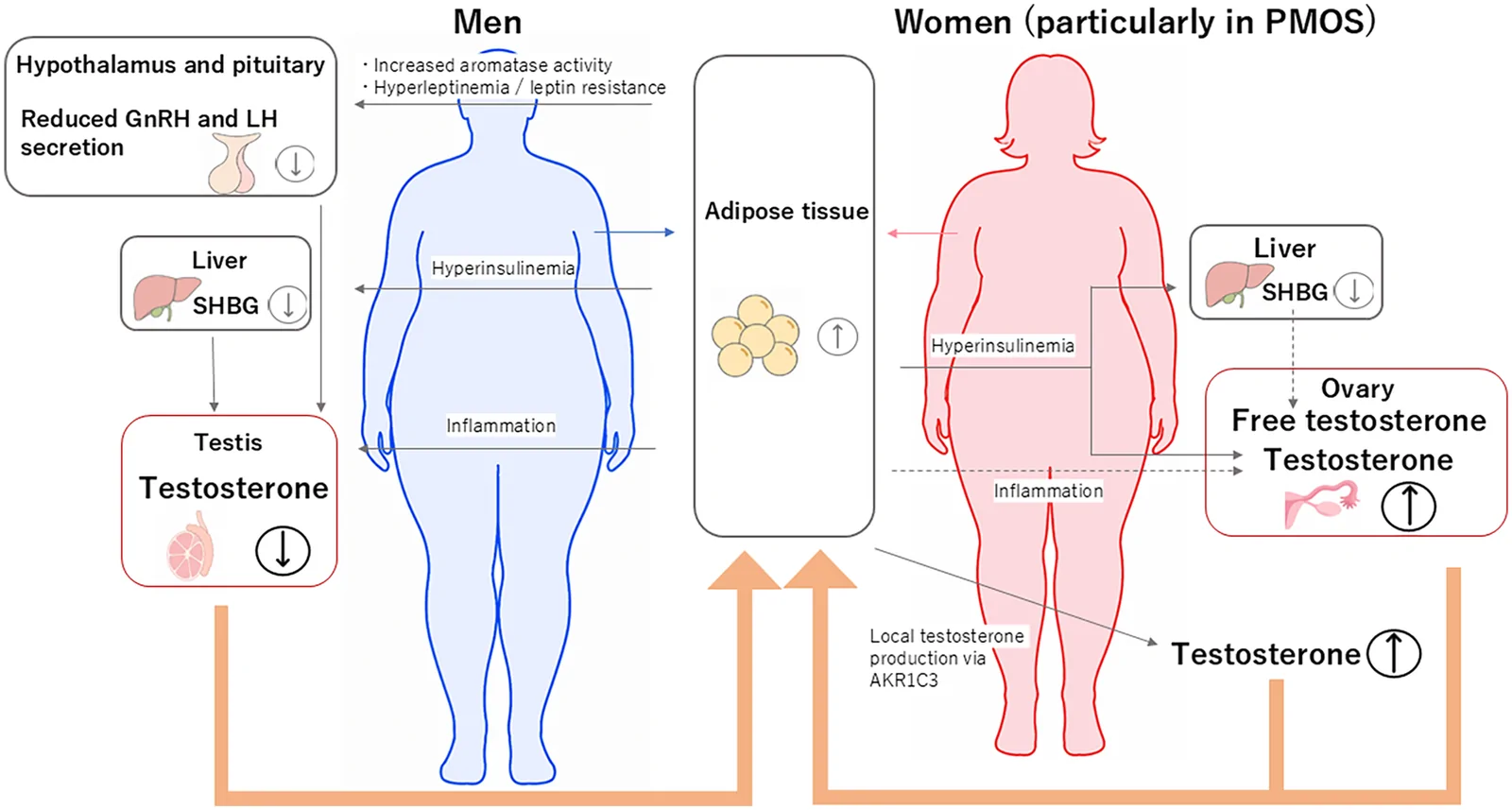
Sex-specific bidirectional relationships between testosterone and obesity. In men, obesity contributes to reduced testosterone through suppression of the HPG axis, decreased SHBG, hyperinsulinemia, and inflammatory signaling, while low testosterone may further promote adipose tissue accumulation. In women, hyperinsulinemia enhances ovarian androgen production and reduces SHBG, thereby increasing testosterone bioavailability. Local androgen production in adipose tissue via AKR1C3 may further contribute to androgen excess and adipose dysfunction. GnRH, gonadotropin-releasing hormone; LH, luteinizing hormone; SHBG, sex hormone-binding globulin; AKR1C3, aldo-keto reductase family 1 member C3; PMOS, polyendocrine metabolic ovarian syndrome.

### 3.3 The vicious cycle of testosterone and obesity

In men, decreased testosterone and obesity have been proposed to form a bidirectional relationship and may influence one another. As discussed, hypogonadism may promote fat accumulation by enhancing adipocyte differentiation and reducing muscle mass. Conversely, obesity may further reduce testosterone production via increased aromatase activity, suppression of the HPG axis, chronic inflammation, and obesity-related OSA. This obesity-associated, potentially reversible form of secondary hypogonadism is commonly referred to as male obesity-related secondary hypogonadism (MOSH) (27). Clinically significant weight loss can increase serum testosterone concentrations, with the magnitude of improvement generally proportional to the degree of weight reduction (3, 5, 27). Therefore, weight loss and optimization of obesity-related comorbidities should form the foundation of MOSH management. Testosterone replacement therapy may be considered in selected men with persistent symptoms and unequivocally low testosterone concentrations after appropriate evaluation, but it should not replace interventions targeting the underlying obesity and associated metabolic disorders (5, 27, 28).

## 4 Interaction between testosterone and obesity in women

### 4.1 Testosterone elevation induced by obesity

In contrast to the reduction in testosterone commonly observed in men, obesity in women may be associated with relative androgen excess (29). Insulin resistance and compensatory hyperinsulinemia are important factors influencing the androgenic environment in women. Insulin enhances ovarian androgen production by augmenting the steroidogenic response to luteinizing hormone. Furthermore, hyperinsulinemia reduces hepatic sex hormone-binding globulin production, thereby increasing the free, biologically active fraction of circulating testosterone (29). In addition, adipose tissue contributes to local androgen metabolism. Aldo-keto reductase family 1 member C3 (AKR1C3), which is expressed in adipose tissue, converts androstenedione to testosterone and thereby regulates local androgen availability. In women with PMOS, adipose AKR1C3-mediated androgen activation has been implicated in fat accumulation and metabolic dysfunction. However, the available evidence is derived mainly from women with PMOS and experimental studies. Thus, local androgen activation in adipose tissue may contribute to relative androgen excess in women with obesity, particularly in those with PMOS (30, 31) (Figure 3). Taken together, obesity-related adipose tissue dysfunction may promote insulin resistance and compensatory hyperinsulinemia. Hyperinsulinemia may then enhance luteinizing hormone-stimulated ovarian androgen production and suppress hepatic SHBG production, thereby increasing free testosterone (29–31). Increased androgen exposure may, in turn, further aggravate adipose tissue dysfunction and insulin resistance, particularly in PMOS (32–36) (Figure 3). Because this evidence is derived mainly from women with PMOS and experimental studies, it should not be generalized to women with obesity who do not have hyperandrogenic disorders.

### 4.2 The impact of a hyperandrogenic environment on obesity and metabolic dysfunction

PMOS is a representative clinical condition in which androgen excess promotes obesity and metabolic dysfunction. Women with PMOS frequently exhibit abdominal adiposity, obesity, insulin resistance, and impaired glucose tolerance (32, 33). The central pathophysiology of PMOS involves excessive ovarian androgen production, which is further amplified by compensatory hyperinsulinemia associated with insulin resistance and obesity (32, 34). Conversely, androgen excess may alter adipose tissue function and promote adverse fat distribution and metabolic dysfunction. Women with PMOS have higher circulating androgen concentrations and a greater android-to-gynoid fat mass ratio than BMI-matched controls. Although subcutaneous adipose AKR1C3 expression is not consistently increased, its expression is associated with fat accumulation in women with PMOS, suggesting a role for local androgen activation in adipose tissue (31). Women with PMOS exhibit adipose tissue dysfunction, including altered adipokine secretion and chronic low-grade inflammation (35). Experimental studies further suggest that androgen excess directly affects adipose tissue through androgen receptor signaling. In a dihydrotestosterone (DHT)-induced mouse model of PMOS, androgen exposure increased adiposity and adipocyte hypertrophy, whereas these changes were absent following adipose tissue-specific androgen receptor deletion (36). These findings suggest that androgen receptor signaling involving adipose tissue contributes to fat accumulation and metabolic dysfunction under hyperandrogenic conditions (36). In summary, PMOS appears to be characterized by a vicious cycle in which obesity and hyperinsulinemia stimulate androgen production, and the subsequent increase in androgens further aggravates fat accumulation, adipose tissue dysfunction, and insulin resistance. However, these insights are primarily derived from studies on PMOS and androgen-excess models. Evidence in women without PMOS or biochemical hyperandrogenism is considered separately in Section 5.3. Thus, experimental androgen-excess models provide mechanistic evidence that androgen receptor signaling in adipose tissue contributes to adipocyte hypertrophy and fat accumulation. In contrast, clinical studies in women with PMOS have mainly demonstrated associations among hyperinsulinemia, reduced SHBG, androgen excess, adverse fat distribution, and insulin resistance; their predominantly observational design limits causal inference.

## 5 Future research directions

### 5.1 Testosterone–estradiol balance and estrogen action in adipose tissue

Testosterone and estradiol exert their effects through the androgen and estrogen receptors, respectively, while testosterone also serves as a substrate for estradiol synthesis through aromatase (CYP19A1). Studies examining the relationship between testosterone and obesity should therefore distinguish the direct effects of testosterone from those mediated through its conversion to estradiol. Adipose tissue expresses aromatase and estrogen receptors and is involved in local sex steroid metabolism and action. A study of individuals with severe obesity demonstrated that the expression patterns of aromatase and estrogen receptors in adipose tissue varied according to sex, age, menopausal status, and adipose tissue depot, including omental and subcutaneous adipose tissue (37). In addition, a study of pre- and postmenopausal women evaluated adipose tissue estrogen receptor expression and estradiol-stimulated glucose uptake in adipocytes, suggesting a relationship between estrogen action and adipose tissue glucose metabolism (38). The metabolic consequences of obesity are also determined not only by body mass index or total fat mass but also by visceral and subcutaneous fat distribution, ectopic fat accumulation, adipose tissue expandability, fibrosis, and inflammation (39). The androgenic environment is not determined by testosterone alone. Dehydroepiandrosterone (DHEA), dehydroepiandrosterone sulfate (DHEA-S), androstenedione, DHT, and 11-oxygenated androgens differ in their origins, metabolism, and biological activities. In particular, 11-ketotestosterone and 11-ketodihydrotestosterone have androgen receptor activities comparable to those of testosterone and DHT and contribute to androgen excess in PMOS (40). Thus, total testosterone alone may not adequately reflect overall androgen action, particularly in women. Circulating concentrations may also differ from local steroid activity because adipose tissue can convert androstenedione to testosterone through AKR1C3, thereby modifying local androgen exposure (30). Future studies should therefore combine liquid chromatography–tandem mass spectrometry (LC-MS/MS)-based measurement of testosterone, estradiol, SHBG, free testosterone, classical and 11-oxygenated androgens with longitudinal assessments of adiposity, fat distribution, insulin resistance, and glucose metabolism (40, 41). Where feasible, circulating profiles should be integrated with intratissue steroid concentrations and steroidogenic enzyme expression in adipose tissue.

### 5.2 Phenotyping and predicting recovery of obesity-related low testosterone in men

Obesity-related low testosterone in men includes reductions in total testosterone driven predominantly by low sex hormone-binding globulin and phenotypes accompanied by reduced free testosterone and functional suppression of the HPG axis (3, 5). However, criteria that reliably distinguish these phenotypes and predict recovery of gonadal function after weight loss have not been established. Future studies should repeatedly assess total testosterone, sex hormone-binding globulin, free testosterone, and gonadotropins and examine their longitudinal relationships with visceral adiposity, hepatic fat, insulin resistance, obstructive sleep apnea, and inflammatory markers. In particular, it remains unclear whether testosterone recovery is determined primarily by the magnitude of total weight loss or by improvements in specific metabolic factors, such as visceral and hepatic fat or insulin sensitivity. Lifestyle intervention, anti-obesity pharmacotherapy, and metabolic bariatric surgery also differ in the rate of weight loss and their effects on body composition and metabolic function. Although testosterone concentrations generally increase after weight loss, studies directly comparing these interventions at equivalent degrees of weight reduction and evaluating long-term changes in total testosterone, free testosterone, sex hormone-binding globulin, and gonadotropins remain limited (42). Prospective comparative studies are therefore needed to identify treatment-specific patterns of gonadal recovery and develop predictive models that distinguish reversible functional suppression from organic hypogonadism.

### 5.3 Testosterone and obesity in women beyond PMOS

Research on androgen action in women has predominantly focused on pathological hyperandrogenism, such as PMOS, and experimental models of androgen excess. Consequently, the metabolic role of androgens within the physiological female range remains insufficiently understood (43). A cross-sectional study in the general female population showed an L-shaped inverse association between total testosterone and metabolic syndrome, although causality and an optimal physiological concentration range remain unknown (44). Evidence specifically addressing the relationship between physiological testosterone concentrations and obesity in women without PMOS remains limited. In healthy reproductive-aged women, circulating testosterone concentrations were inversely associated with BMI and were higher in normal-weight women than in overweight women (45). These findings raise the possibility that relatively low testosterone concentrations within the physiological female range may also be associated with an unfavorable adiposity-related phenotype (45, 46). However, this hypothesis has not been established in longitudinal human studies, and it remains unclear whether lower testosterone contributes to fat accumulation or primarily reflects hormonal and metabolic changes associated with increased adiposity. Future longitudinal studies should repeatedly assess total testosterone, free testosterone, SHBG, estradiol, menopausal status, insulin resistance, and detailed measures of body composition in women without PMOS. Such studies are needed to determine whether physiological testosterone concentrations predict subsequent changes in body weight, visceral adiposity, and fat distribution and to clarify whether their associations with obesity are independent of SHBG and other metabolic factors.

## 6 Conclusion

Testosterone and obesity interact bidirectionally, but the nature and metabolic significance of this relationship differ markedly between men and women. In men, obesity commonly lowers circulating testosterone through reductions in sex hormone-binding globulin and, in some individuals, functional suppression of the HPG axis. Reduced androgen action may then promote fat accumulation, loss of skeletal muscle, and metabolic dysfunction, reinforcing the relationship between obesity and low testosterone. In women, obesity-related insulin resistance and hyperinsulinemia may increase ovarian androgen production and androgen bioavailability, whereas pathological androgen excess, particularly in PMOS, may further aggravate adverse fat distribution, adipose tissue dysfunction, and insulin resistance. However, findings derived from PMOS and androgen-excess models should not be extrapolated directly to androgen action within the physiological female range. Experimental and observational findings raise the possibility that physiological androgen receptor signaling contributes to metabolic homeostasis, whereas excessive activation is detrimental. Overall, testosterone cannot be regarded as uniformly beneficial or harmful. Its metabolic effects depend on sex, hormone concentration, bioavailability, conversion to other sex steroids, target tissue, and the surrounding metabolic environment. Recognition of these differences is essential for understanding the sex-specific relationship between testosterone and obesity and for developing more precise approaches to obesity-related metabolic disease.
